# SPALEX: A Spanish Lexical Decision Database From a Massive Online Data Collection

**DOI:** 10.3389/fpsyg.2018.02156

**Published:** 2018-11-12

**Authors:** Jose Armando Aguasvivas, Manuel Carreiras, Marc Brysbaert, Paweł Mandera, Emmanuel Keuleers, Jon Andoni Duñabeitia

**Affiliations:** ^1^BCBL, Basque Center on Brain, Language and Cognition, Donostia, Spain; ^2^Ikerbasque, Basque Foundation for Science, Bilbao, Spain; ^3^Department of Experimental Psychology, Ghent University, Ghent, Belgium; ^4^Department of Cognitive Science and Artificial Intelligence, Tillburg University, Tillburg, Netherlands; ^5^Facultad de Lenguas y Educación, Universidad Nebrija, Madrid, Spain

**Keywords:** megastudies, lexical decision, vocabulary knowledge, online assessments, lexical database

## SPALEX^1^ a spanish lexical decision database from a massive online data collection

“Megastudy” is the term coined to refer to large-scale experiments completed by thousands (or even hundred-thousands) of participants (Chetail et al., 2015; Gimenes et al., 2016). These types of studies have exponentially increased in the last decade, from around 130 publications referencing the term in 2007 to more than 300 in 2017 (Kuperman, 2015). Their uses span different fields, such as cognitive science, medicine, education, and psychology research. Particularly for the field of psycholinguistics, the increasing demand for massive and diverse databases upon which non-trivial hypotheses and models can be tested, has led to a dramatic increase in large-scale lexical megastudies (Keuleers and Balota, 2015).

Lexical decision megastudies have been carried out in multiple languages, including American and British English ((Balota et al., 2007; Keuleers et al., 2012); respectively), French (Ferrand et al., 2010), and Dutch (Keuleers et al., 2010; Brysbaert et al., 2016). In Spanish, previous laboratory studies have explored the effects of psycholinguistic variables on a large amount of words, but with a relatively small number of participants (Davies et al., 2013; González-Nosti et al., 2014). Although these are laboratory studies that involve a large number of participants, or a large number of words, other approaches, such as crowdsourced megastudies, distributed through online platforms, allow the collection of information with large numbers of participants and words at a reduced cost (Keuleers et al., 2015).

The shift in view from the laboratory to this type of crowdsourced research makes novel technologies like smartphones or tablets powerful research tools that allow for large-scale studies (Dufau et al., 2011). Likewise, large-scale studies have the benefit of studying and quantifying phenomena of interest across a varied and a vast number of participants. Another essential advantage of megastudies is the ability to conduct virtual experiments with randomly selected samples within the same database to test multiple hypotheses of different nature (Kuperman, 2015). They are becoming essential for psycholinguistic studies, as the study of language has been traditionally focalized in small and homogeneous groups of participants, not allowing the evaluation of important factors, such as previous linguistic experience, degree of second-language proficiency, or age, to cite a few (Keuleers et al., 2015).

In all, megastudies provide a robust framework to test theories and provide important information that can be used for further experimentation. So far, no previous attempt has been made to produce a crowdsourced lexical decision megastudy in Spanish, which with about 400 million speakers across the world (Ethnologue, 2016), is the second most used native language after Chinese. A well-designed study would also allow highlighting differences in how Spanish is used in the more than 20 Spanish-speaking countries across the globe. Moreover, the database presented here, henceforth called SPALEX, adds to the increasing literature on lexical decision megastudies by focusing on native Spanish speakers at a global scale and with a vast amount of words, to provide a useful tool for researchers exploring the acquisition and processing of this language in native and foreign contexts.

## The SPALEX database

SPALEX contains data from a Spanish crowdsourced lexical decision megastudy. We collected the data through an online platform from May 12th, 2014 to December 19th, 2017. Up to that point, 209,351 participants had finished 282,576 tests by completing one (80.01%), two (14.11%), three (3.28%), or more sessions (2.60%). The majority of the data (68.88%) was acquired during the first month of the experiment, when an advertising campaign was done in order to attract the public's attention. Participants also had the option of publishing their results via social networks, which led to attract more participants in a snow-ball sampling fashion.

Additionally, the database contains information on participants that voluntarily provided information about their gender, age, country of origin, education level, handedness, native language, and best foreign language. A significant percentage of participants voluntarily provided all of the requested information (79.58%). Of those, 44.66% were females, while 9.02% provided no gender information.

**Figure 1:**
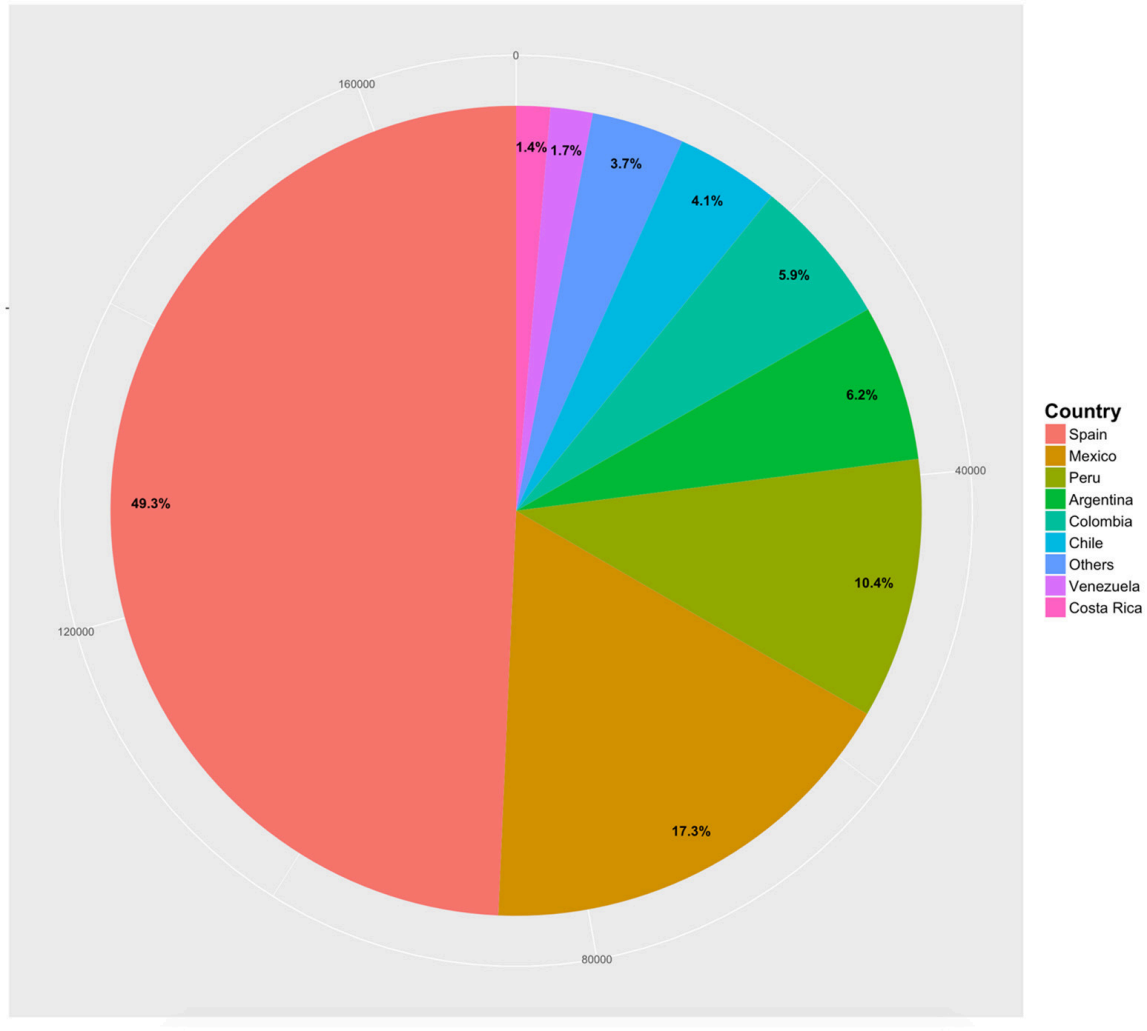
Distribution of participants per country. Countries representing less than one percent of the data were combined into the ‘Others' category. These are: Panama (0.1%), Honduras (0.1%), Nicaragua (0.1%), Paraguay (0.2%), Bolivia (0.2%), Cuba (0.2%), El Salvador (0.2%), Dominican Republic (0.2%), Guatemala (0.4%), Uruguay (0.9%), and Ecuador (0.9%).

We created recoded demographic variables and included them in the final database to facilitate its usage. One important example is the recoded variable of location (location_rec), that was constructed based on the country of origin of each participant, to identify those born in Spain and those born in Latin America. Participants that did not belong to any of those categories were not included in the database (17.44% of the data), as the focus of this database is on native Spanish speakers. After this, a total of 169,628 participants that completed 227,655 sessions remained. Figure 1 shows the distribution of native Spanish speakers per country of origin, indicating that approximately half of the sample was born in Spain, while the remaining half was born in a Latin-American country.

### Lexical decision

In each experimental session, participants responded to 70 words and 30 non-words presented randomly and without repetition. Their accuracy and reaction times were automatically recorded. The task was not speeded, so participants could take all the time they needed to respond to an item. The total amount of items was chosen to ensure that each session would last around 5 min, so that participants wouldn't be discouraged to participate. During the task, they were asked to indicate for each letter string whether they knew the word or not, and they were told that some letter strings were nonce words for which they would be penalized if they selected them as known words. The procedure was analogous to that reported in Keuleers et al. (2015).

The 70/30 word-to-non-word ratio was selected to warrant that more information would be collected from words rather than non-words, and to minimize the effect of words with very low frequency that could be also be regarded as non-words. To ensure that there was no a priori bias in the list composition that could impact the responses, we used the LD1NN algorithm (Keuleers and Brysbaert, 2011). This procedure returns the odds of selecting words over non-words within a lexical decision task (e.g., a ratio of 3 indicates that LD1NN is 3 times more likely to select words over non-words) and has been tested in previous large-scale lexical decision tasks, producing results that range from 0.34 to 4.1 (Keuleers and Brysbaert, 2011). We performed iterations of the algorithm in a randomly selected subsample of 500 participants from our database. Results showed a mean of 2.6 (*SD* = 0.36), which fits well with the values reported in preceding studies despite not using the traditional 50/50 word-to-non-word ratio.

The items were randomly selected from a stimulus list of 45,389 Spanish words and 56,855 non-words. To create the word list, several words were obtained from the B-PAL (Davis and Perea, 2005) and the EsPal databases (Duchon et al., 2013) to account for both written and subtitle-based corpora. From this initial list, we discarded proper nouns and inflected forms of nouns, verbs and adjectives, as well as other compound words, to maximize the information provided by the base form of words in Spanish. This entire list was then fed to Wuggy (Keuleers and Brysbaert, 2010; freely available at http://crr.ugent.be/programs-data/wuggy) to generate several potential non-word candidates for each word. The resulting list (260, 252 non-words) was then put through a lemmatizer to remove inflected forms of existing base forms, and a subset of the list was selected based on the candidate index produced by Wuggy (non-words with a lower index were considered better candidates).

Accuracy in SPALEX is expressed as 1 for correct answers and 0 for incorrect answers. Cronbach's alpha for accuracy scores is 0.76. Average accuracy for the database was 0.79. Response times (RTs) are expressed in milliseconds. After trimming RTs for correct responses between 200 and 2,000 ms (as in (Ferrand et al., 2010)), and removing outliers above and below 1.5 box lengths, average RT was 1,062 ms (*SD* = 362), with a mean of 1,003 ms (*SD* = 348) for words, and 1,198 ms (*SD* = 358) for non-words. While these RTs are longer than those presented in preceding laboratory studies in Spanish using a speeded lexical decision task (e.g., González-Nosti et al., 2014), a series of analyses demonstrated the internal consistency and reliability of the current dataset.

Split-half reliabilities for the RT measure were calculated using the *splithalf* package in R version 3.5.1 for Windows, which is specifically designed for chronometric studies. Because of the large amount of data, we opted to feed the data via batches of 500 randomly selected participants and to run 1,000 iterations of random splits, taking into consideration the accuracy and lexical status (word vs. non-words) of the items. The average split-half correlation score for words was *r* = 0.86 (*SD* = 0.017, range = 0.76–0.91) and *r* = 0.84 (*SD* = 0.012, range = 0.80–0.88) for non-words. Using the Spearman-Brown formula we obtained an average of *r*_*corr*_ = 0.92 (*SD* = 0.009, range = 0.87–0.95) for words and *r*_*corr*_ = 0.91 (*SD* = 0.007, range = 0.89–0.93) for non-words.

Based on participants' responses, we calculated percentage known, a measure of the percentage of participants that know a particular word (Brysbaert et al., 2016). This measure was calculated for the entire database, with around 333 observations per word (*SD* = 77.86), and separately for Spanish speakers from Spain (*M* = 170.08, *SD* = 42.79 observations per word) and Latin-America (*M* = 163.01, *SD* = 39.20 observations per word). Words with a total number of observations below 150 (0.68%) or higher than 1,500 (0.15%) were identified as outliers and removed from the database, leaving a total of 44,853 words. The mean of the percentage known for the total word database was 75.74 (*SD* = 27.00), and it was somewhat larger for Spanish speakers from Spain (76.52, *SD* = 28.13) than from Latin-America (72.88, *SD* = 27.62).

Brysbaert et al. (2016) also introduced the variable word prevalence, which is based on the percentage of language users who know a word. To avoid compression of high values, a probit transformation is usually applied to this percentage, i.e., the inverse of the cumulative normal distribution of the percentage known. This results in prevalence z-scores and allows for some straightforward interpretations: positive scores indicate that the word is known by more than 50% of the participants; negative scores indicate that < 50% know the word; values between 0 and 2 indicate words that are known to 50–98% of participants; a value near 2.5 indicates that nearly everyone knows the word. For the data used in the current study, the mean prevalence z-score was 0.98 (*SD* = 1.04), 1.12 for Spanish natives (*SD* = 1.14), and 0.90 for Latin-American natives (*SD* = 1.05).

**Figure 2:**
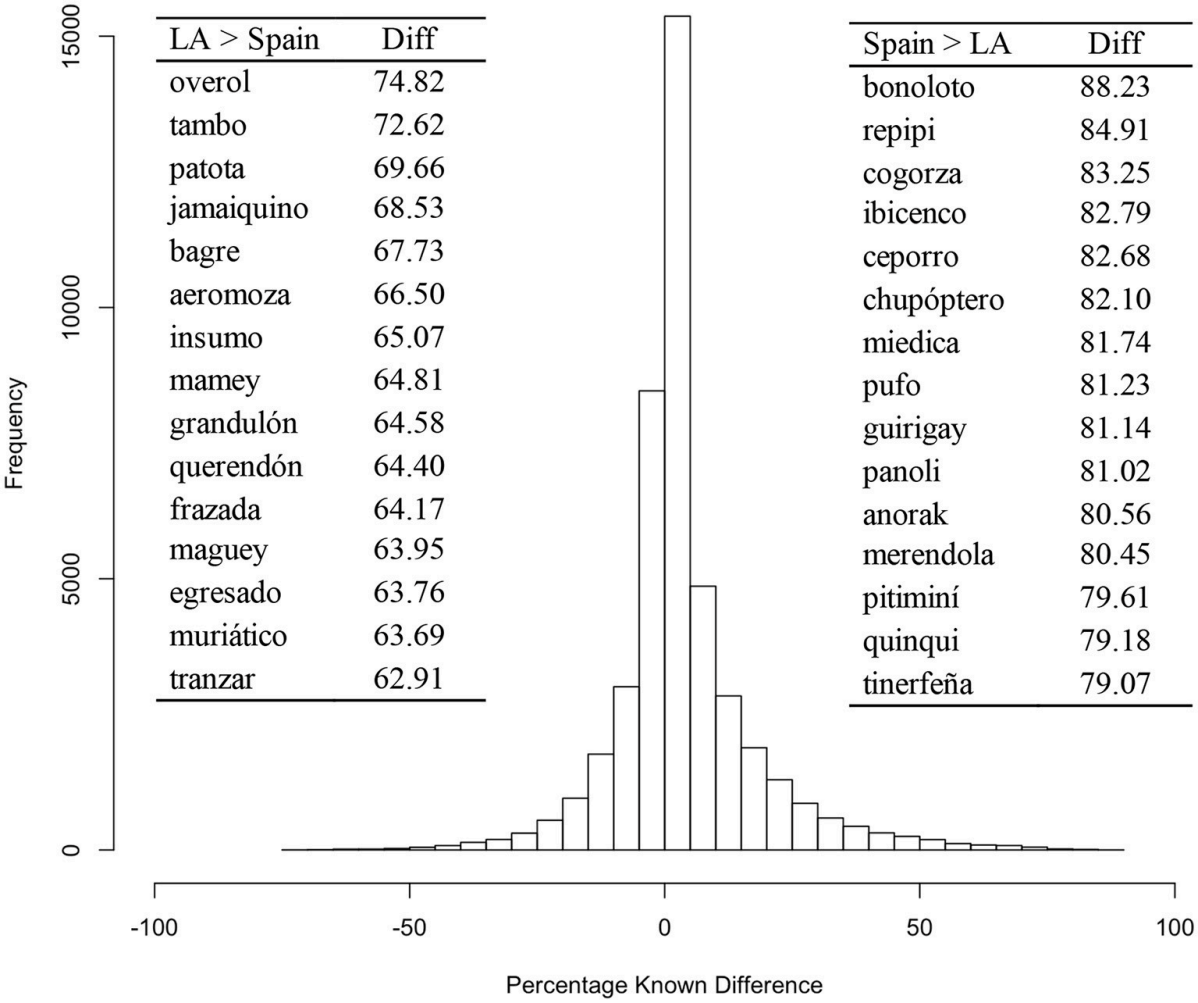
Histogram of the percentage known difference of each word between Spanish and Latin-American natives. The fifteen words with the highest differences for each group are also shown to the right and left of the histogram. LA, Latin-America; Diff, Absolute percentage known difference.

Finally, word frequencies were obtained and incorporated into the database using EsPal. Mean frequency per million was 9.48 (*SD* = 204.41), corresponding to a mean Zipf frequency (Van Heuven et al., 2014) of 2.40 (*SD* = 1.14). Figure 2 shows a histogram of the difference in prevalence between Spanish and Latin-American natives for each word. This figure illustrates that, although the largest percentage of words is known for both groups (70.69% of words diverge in < 10% known points), there are some cultural differences in the usage of vocabulary across regions, as could be expected given the dialectal variations.

## SPALEX files

The following files are contained in the SPALEX database. We opted for uploading the files separately, as unifying them would have led to redundant information and an increased file size. Nevertheless, an R script is included to create a unified file in case it is needed. All the files can be accessed via https://figshare.com/projects/SPALEX/29722

- **users.csv:** Contains demographic information of each participant. Columns are: profile_id (unique identifier), gender (indicated with upper and lowercase m or f), age (M = 46.3, SD = 13.18), country, education (education level from primary school to PhD level), no_foreign_lang (number of foreign languages, M = 2.57, SD = 1.39), best_foreign (best foreign language), handedness (right or left handed), and includes recoded variables for gender (1 = male, 2 = female; gender_rec), education_rec (6 levels), location_rec (2 levels), handedness_rec (2 levels).
- **sessions.csv:** Used to establish the relationship between users (participants) and sessions of the lexical decision task. Columns are: exp_id (unique identifier), date, profile_id.
- **lexical.csv:** Participants' responses to each item in the lexical decision task. Columns are: trial_id (unique identifier), exp_id, trial_order, spelling (presented stimuli), lexicality (word or non-word), rt (reaction time in milliseconds), accuracy (0 or 1), trial_no (trial number from 1 to 100), bins (bins for each two trials, from 1 to 50).
- **word_info.csv:** Information on word percentage known, prevalence, and frequency. Columns are: spelling (unique text identifier), count_total, percent_total (total percentage known), prevalence_total (prevalence scores for the total sample), count_nts^2^, percent_nts, prevalence_nts, count_ntl, percent_ntl, prevalence_ntl, freq (frequency per million), zipf (zipf frequency). This is the file people will find most useful if they simply want information about word knowledge in Spain or Latin-America.
- **merge_script.R:** Script in R language to load the databases and merge them into an unique file. This script may be modified and extended as needed.

## Interpretation and usage

Although the aim of this Data Report is not to provide a comprehensive discussion of the multiple analyses that could be done on these data, we believe that it would be useful for other researchers to understand the real value of SPALEX in relation to other similar databases in different languages. Previous megastudies have measured the effects of different factors on lexical variables. Examples of this are the reported effects of age, education level, and multilingual status on vocabulary size (Keuleers et al., 2015), the impact of word visual complexity in word recognition (Dufau et al., 2015), and the differential processing of singular and plural nouns in various languages (Gimenes et al., 2016), among others (see Keuleers and Balota, 2015, for an overview). The present database could be used in the aforementioned fashion to also account for the Spanish language, but it could be used for other, more general purposes outlined below too.

The first major application of SPALEX relates to the construction of normative data based on gender, age, country of origin, and education level. This would broaden the information already contained in other Spanish lexical databases, allowing extracting information of, for example, the performance of specific age groups on certain words. This is particularly useful for the creation of vocabulary tests. Word prevalence is an understudied measure of word knowledge that is not much correlated to word frequency and has a high impact on word processing, making it a novel approach to prevent bias in stimulus selection (Keuleers et al., 2015; Brysbaert et al., 2016).

Other uses extend both within and outside the bounds of SPALEX. The former is the construction of “mini-experiments” within the database to develop valid working hypotheses of the Spanish language (see Kuperman, 2015, for an example). An example of this is the comparison of vocabulary size depending on the education background of different Spanish speaking countries. The latter involves making cross-linguistic comparisons between this and other similar lexical decision databases in different languages. Myers (2016) states that the fundamental benefit of these “meta-megastudies” is the reduction of language-specific confounds, which in turn allows researchers to draw insights on human language processing itself.

Overall, the ultimate goal of lexical megastudies is to increase the tools available for psycholinguists to develop accurate models of language processing (Keuleers and Balota, 2015; Mandera et al., 2017). In this regard, SPALEX offers information on word processing across multiple Spanish speaking countries, making it the largest lexical decision database in Spanish to date.

## Ethics statement

This study was carried out in accordance with the recommendations of the Ethics Committee of the BCBL. The protocol was approved by this same Committee. All subjects were informed about the nature of the task prior to its completion, and no personal information that could identify any participant was collected. The task was correctly explained once participants voluntarily and anonymously accessed the platform, and they gave informed consent about their participation via button clicks. The protocol mimicked that previously reported in Brysbaert et al. (2016) and Keuleers et al. (2015), among others.

## Author contributions

MC, JD, MB, and EK devised the project and the main conceptual ideas. JD selected the materials in collaboration with PM and EK and PM, EK and MB developed the platform. JA developed the analysis routine under the supervision of JD and MC and all authors verified the analytical methods. All authors supervised the findings of this work. JA drafted the manuscript and all authors discussed the results and contributed to the final manuscript.

### Conflict of interest statement

The authors declare that the research was conducted in the absence of any commercial or financial relationships that could be construed as a potential conflict of interest. The reviewer VK declared a past co-authorship with one of the authors, MB.

## Footnotes

^1^**Access to the database**: https://figshare.com/projects/SPALEX/29722

^2^NTS (Natives from Spain), NTL (Natives from Latin-America)
